# On the Management of Drug Interactions in the Course of Concomitant Treatments for COVID-19 and Antineoplastic Agents

**DOI:** 10.3389/fonc.2020.01340

**Published:** 2020-07-21

**Authors:** Nicola Silvestris, Antonio Munafò, Oronzo Brunetti, Chiara Burgaletto, Luisa Scucces, Renato Bernardini

**Affiliations:** ^1^Medical Oncology Unit, IRCCS Istituto Tumori “Giovanni Paolo II”, Bari, Italy; ^2^Department of Biomedical Sciences and Human Oncology, University of Bari “Aldo Moro”, Bari, Italy; ^3^Section on Pharmacology, Department of Biomedical and Biotechnological Sciences, University of Catania School of Medicine, Catania, Italy

**Keywords:** cancer therapy, Sars-Cov2, drug-to-drug interaction, metabolism, dose adjustement

## Introduction

The unprecedented global pandemic caused by coronavirus 2 (SARS-Cov2) drew medical attention toward patients with co-morbid disorders, who are more exposed to prognostically unfavorable outcomes (1). A peculiar clinical scenario is represented by oncologic patients, who appear more susceptible to COVID-19 infection, as they may develop more severe symptoms than those observed in individuals with different comorbidity profiles (2).

## Background and Rationale

Comorbid patients require multiple pharmacological therapies, which may, in turn, result in issues that Clinicians are asked to address quickly by considering the possible drug-drug interactions that may occur, with the aim of preventing reduced effectiveness or increased burden of adverse events (3). Generally speaking, the question of whether concomitant pharmacological therapies may threaten the safety of patients is usually answered in a context that acknowledges the treatment options for each single disease, allowing fair management of interactions on the basis of robust clinical evidence (4). On the other hand, in case of comorbidities occurring in COVID-19 patients, Physicians are now asked to answer the challenging question of whether interactions are possible between pharmacological treatments for COVID-19, which are not well-defined yet, and antineoplastic agents (5). In fact, whilst awaiting results from over 300 clinical trials currently underway, which aim to identify effective therapies against the COVID-19 syndrome, how drugs used for COVID-19 patients (e.g., hydroxychloroquine, antiviral agents, monoclonal antibodies) (6) may redundantly influence the pharmacokinetics and pharmacodynamics of cancer drugs (e.g., chemotherapy, hormonotherapy, targeted therapy, and immunotherapy) remains an object of investigation (7). This issue is crucial interest in patients bearing a cancer who are co-morbid for COVID-19, while remaining asymptomatic or paucisymptomatic. In these patients, a delay in the administration of a scheduled oncological treatment may have a meaningful impact on both survival and quality of life outcomes (8).

Attention should thus be focused on the interactions of the drugs most commonly used for COVID-19 with different classes of antineoplastic drugs (Table 1).

**Table 1:**
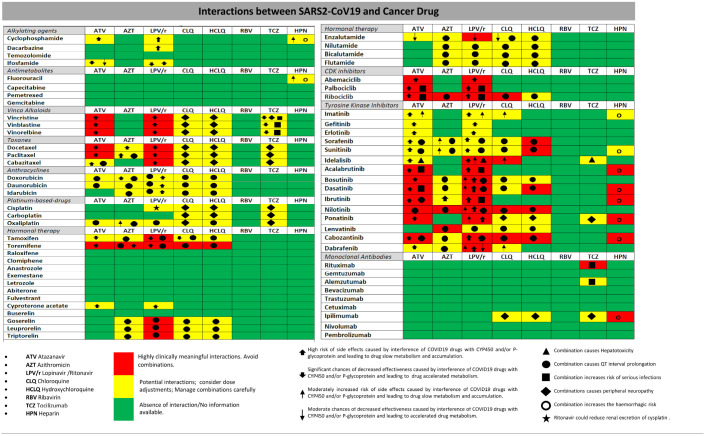
Checker illustrating likely interactions between antitumor agents and the drugs actually used in the SARS-Cov2 infection.

*Interactions, which require major attention (red color), may occur between antiretroviral agents and chloroquine/hydroxychloroquine with Vinca alcaloids, Taxanes, Anti-estrogens, as well as CDK and TK inhibitors. Abbreviations and explanations in the lower part of the table. All checker's matches between drugs were verified in the current literature (9–11)*.

## Discussion

In light of the array of possible interactions with systems of hepatic metabolism, such as cytochrome p450 (CYP450), as most of the actual antiviral agents used in COVID-19 infection are likely to impact on different CYP450 isozymes, dose adjustments of various drugs may be required for either cancer or SARS-Cov2 infection treatments. In fact, respective areas under the curve may be significantly different than expected in case of concomitant administration (12, 13). For example, either atazanavir or the combination lopinavir/ritonavir requires significant dose reduction of CDK4/6 inhibitors, and a full dose may be re-administered after 3–5 half-lives (14). With the same agents, administration of chloroquine should be intensely monitored because of the increased risk of QTc prolongation (15, 16). Also P-glycoprotein, a member of the ABC superfamily, regulating the efflux of drugs from cells, and similarly the multidrug and toxin extrusion protein-1 (MATE-1) are involved as targets for significant drug-drug interactions related to the administration of platinum compounds (17, 18). Thus, decreased efficacy and increased rates of resulting grade 3/4 adverse events (e.g., peripheral neuropathy, QT prolongation, increased risk of infection) need to be constantly and carefully addressed during concomitant anti-COVID-19-tumor treatments (19, 20). Thus, measurement of plasma/serum drug concentrations in the course of treatment appears an opportune procedure to avoid penalizing the efficacy of antiblastic treatments or exacerbation of toxicity. Interactions appear more likely to happen when using chemotherapics and/or hormonal therapy and may jeopardize the relatively satisfying results obtained, for example, in diseases such as the hormone dependent breast cancer or prostate cancer, which highly benefit of actually available treatments in terms of survival and quality of life.

In order to achieve a better knowledge on the management of different cancer treatment schedules in COVID-19 patients without compromising efficacy and safety, *ad hoc* observational clinical studies should be implemented with proper clinical endpoints pointing to the dose adjustments needed in case of emerging interactions.

## Author Contributions

NS and RB: manuscript conception, writing, and revision. OB, CB, LS, and AM: manuscript elaboration and writing. All authors contributed to the article and approved the submitted version.

## Conflict of Interest

The authors declare that the research was conducted in the absence of any commercial or financial relationships that could be construed as a potential conflict of interest.
